# Hierarchical Cosine Similarity Entropy for Feature Extraction of Ship-Radiated Noise

**DOI:** 10.3390/e20060425

**Published:** 2018-06-01

**Authors:** Zhe Chen, Yaan Li, Hongtao Liang, Jing Yu

**Affiliations:** 1 School of Marine Science and technology, Northwestern Polytechnical University, Xi’an 710072, China; 2 School of Physics and Information Technology, Shaanxi Normal University, Xi’an 710119, China

**Keywords:** hierarchical cosine similarity entropy, multiscale entropy, sample entropy, feature extraction, complexity

## Abstract

The classification performance of passive sonar can be improved by extracting the features of ship-radiated noise. Traditional feature extraction methods neglect the nonlinear features in ship-radiated noise, such as entropy. The multiscale sample entropy (MSE) algorithm has been widely used for quantifying the entropy of a signal, but there are still some limitations. To remedy this, the hierarchical cosine similarity entropy (HCSE) is proposed in this paper. Firstly, the hierarchical decomposition is utilized to decompose a time series into some subsequences. Then, the sample entropy (SE) is modified by utilizing Shannon entropy rather than conditional entropy and employing angular distance instead of Chebyshev distance. Finally, the complexity of each subsequence is quantified by the modified SE. Simulation results show that the HCSE method overcomes some limitations in MSE. For example, undefined entropy is not likely to occur in HCSE, and it is more suitable for short time series. Compared with MSE, the experimental results illustrate that the classification accuracy of real ship-radiated noise is significantly improved from 75% to 95.63% by using HCSE. Consequently, the proposed HCSE can be applied in practical applications.

## 1. Introduction

Ship-radiated noise is the main signal source of passive sonar for underwater target detection and recognition. Extracting useful features from ship-radiated noise can effectively improve the performance of passive sonar. Thus, the research of feature extraction techniques has received considerable attention [1,2,3].

The propulsion parts of a ship, such as engines, turbines and propellers, are the principal sources of ship-radiated noise [4]. The emitted noise is propagated in the ocean channel and eventually received by hydrophones. Because of the complicated production mechanism and the influence of stochastic ocean medium, the received ship-radiated noise is usually nonstationary, non-Gaussian and nonlinear. It is challenging to extract useful features from such a signal. The physical features, such as the blade rate, propeller shaft frequency and the number of blades, have been studied in past decades by utilizing frequency domain based techniques, such as the power spectrum density (PSD), short-time Fourier transform (STFT) and wavelet transform [5,6,7,8]. These traditional feature extraction methods have achieved great effectiveness in practical engineering applications, but there are still some limitations. For example, the PSD is not able to reflect local properties of a signal, while the wavelet transform is limited by the selection of wavelet basis function, improper choice of wavelet basis function may lead to distortion [3,7]. Moreover, physical features are not sufficient to classify ships that have similar propulsion components [1].

Although ship-radiated noise is nonlinear, this characteristic is neglected by traditional linear processing techniques. Therefore, there is a growing demand to extract the nonlinear features of ship-radiated noise [9]. Entropy is a powerful nonlinear analysis tool that can analyze complex mechanical systems [10,11,12]. To date, various statistical entropy algorithms for quantifying the complexity of signals have been developed, such as permutation entropy [13,14], approximate entropy (ApEn) [15] and sample entropy (SE) [16]. One of the most commonly used structural complexity estimators, SE, which is obtained by calculating the conditional probability of occurrences of similar patterns, has attracted considerable attention in numerous fields such as biological signal analysis [17], fault diagnosis [18] and acoustic signal processing [9]. However, despite its great success, there exist some drawbacks: (I) High computational cost. Calculating SE needs to estimate the probability of occurrence of similar patterns found in the reconstructed m and (m + 1) dimensional phase-space, any two vectors in the phase-space are similar if their Chebyshev distance is lower than a predefined tolerance $r_{SE}$. The similarity checking can result in the computation cost quadratically increasing as the data-length increases [19], which may limit the SE’s performance in some real-time applications; (II) Sensitive to erratic noise [20]. In SE, the similarity checking is based on the Chebyshev distance, which is amplitude dependent, leading to that high peaks existing in the time series can directly affect the entropy estimates. Unluckily, the unwanted erratic noise is likely to present in real data. For example, when a hydrophone is recording the underwater sound, it is unavoidable to be influenced by waves, which will produce undesirable outliers; (III) Undefined entropy value. The SE is effective when data-length is more than $10^{m}$ [21], where m denotes the embedding dimension. However, as the data-length decreases, SE may produce undefined entropy value because of few similar patterns existing in the reconstructed m and (m + 1) dimensional phase-space [22]; (IV) Single-scale based [16,19,20,21]. Since complex signals often show structures on multiple temporal scales, the SE method is not able to estimate structural complexity of such a time series comprehensively and precisely.

In order to overcome the above mentioned shortcomings, many efforts have been made. Recently, there have been some solutions to compute SE quickly [19,23,24]. Furthermore, cosine similarity entropy (CSE) [20] was proposed to deal with issue (II) and (III), whereby the angular distance is employed instead of the Chebyshev distance, and the conditional entropy is replaced with the Shannon entropy. Regarding the fourth issue, the multiscale entropy (MSE) was introduced by Costa et al. [25,26,27] to measure complexity over a range of temporal scales. The method shows that the entropy of white noise decreases as the scale factor increases, which agrees with the fact that the white noise is not structurally complex. Therefore, the MSE algorithm offers a better interpretation of the complexity of a signal. Even though, the MSE only takes the lower frequency components of a time series into consideration, while the higher frequency components are ignored [28]. To remedy this, a new multiscale decomposition technique, the hierarchical decomposition was developed by Jiang et al. [28]. By further combining the hierarchical decomposition with SE, the scheme (i.e., hierarchical sample entropy (HSE)) is capable of analyzing a signal more adequately than MSE. Unfortunately, since SE is not perfect, its limitations are bound to be retained in HSE.

In this paper, the hierarchical cosine similarity entropy (HCSE) is proposed for feature extraction of ship-radiated noise. The presented algorithm takes advantages of both hierarchical decomposition and CSE. By analyzing synthetic signals, a set of parameters for computing the HCSE is recommended. The simulation results indicate that HCSE overcomes some limitations in SE and MSE. By employing HCSE to extract features of experimental data (see Section 4.2 for detailed description), the classification performance is significantly improved. The remainder of this paper is organized as follows: Section 2 provides a description of the proposed HCSE; parameter selection for HCSE is studied in Section 3; the HCSE is employed to analyze synthetic signals and experimental data in Section 4; the paper is concluded in Section 5.

## 2. Hierarchical Cosine Similarity Entropy

### 2.1. Cosine Similarity Entropy

For a time series ${\left\{x_{i}\right\}}_{i=1}^{N}$, the CSE is computed as follows:

(1) Given embedding dimension m and time delay τ, the embedding vectors are constructed as:

(1) $$x_{i}^{\left(m\right)}=\left[x_{i},x_{i+\tau },\cdots ,x_{i+(m-1)\tau }\right]$$

(2) Calculate angular distance for all pairwise vectors. The angular distance is derived from the cosine similarity and cosine distance. The cosine similarity of two diverse vectors $x_{i}^{\left(m\right)}$ and $x_{j}^{\left(m\right)}$ is defined as:

(2) $$CosSim_{i,j}^{\left(m\right)}=\frac{x_{i}^{\left(m\right)}\cdot x_{j}^{\left(m\right)}}{\left|x_{i}^{\left(m\right)}\right|\cdot \left|x_{j}^{\left(m\right)}\right|}.$$

Notice that the value of cosine similarity is ranging from −1 to 1, the cosine distance is then defined as $CosDis_{i,j}^{\left(m\right)}=1-CosSim_{i,j}^{\left(m\right)}$ to provide a positive distance metric. However, both the cosine similarity and the cosine distance violate the triangle inequality property, neither of them is a proper distance metric [20]. The angular distance, which is defined as $AngDis_{i,j}^{\left(m\right)}=arccos(CosSim_{i,j}^{\left(m\right)})/\pi$, obeys the axioms of a valid distance metric and thus be an appropriate selection for measuring distance of vectors. It is deserved to mention that the distance metric is one of the differences between SE and CSE. In SE, the Chebyshev distance is used for quantifying distance of vectors, which can be written as $CheDis_{i,j}^{\left(m\right)}=max(\left|x_{i}^{\left(m\right)}(k)-x_{j}^{\left(m\right)}(k)\right|)$, k=1,2,⋯,m. It can be concluded that the Chebyshev distance is amplitude based and sensitive to outliers, while the angular distance is more stable.

(3) Given a tolerance $r_{CSE}$, any two diverse vectors $x_{i}^{\left(m\right)}$ and $x_{j}^{\left(m\right)}$ are regarded as similar patterns if $AngDis_{i,j}^{\left(m\right)}\leq r_{CSE}$. Count the number of similar patterns of each vector $P_{i}^{\left(m\right)}$, the local and global probability of occurrences of similar patterns can be calculated as:

(3) $$A_{i}^{\left(m\right)}=\frac{1}{N-m}P_{i}^{\left(m\right)},$$

(4) $$A^{\left(m\right)}=\frac{1}{N-m+1}\sum_{i=1}^{N-m+1}A_{i}{}^{\left(m\right)}.$$

(4) The CSE is finally defined in the form of Shannon entropy:

(5) $$CSE=-\left[A^{\left(m\right)}{log}_{2}\left(A^{\left(m\right)}\right)+\left(1-A^{\left(m\right)}\right){log}_{2}\left(1-A^{\left(m\right)}\right)\right].$$

By employing Shannon entropy to estimate CSE, the entropy values of CSE range from 0 to 1. It means that a time series is structurally complex when CSE approaches 1 and structurally simple when CSE approaches 0. Unlike SE, undefined entropy value is unlikely to occur in CSE unless $A^{\left(m\right)}=0$, which means that none vectors are similar.

### 2.2. Hierarchical Decomposition

The MSE algorithm only takes the lower frequency components of a time series into consideration, while the higher frequency components are ignored. To remedy this, the hierarchical decomposition was introduced by Jiang et al. [28]. For a time series ${\left\{x_{i}\right\}}_{i=1}^{N}$, where $N=2^{n}$, it can be decomposed by following procedures:

(1) Averaging operator $Q_{0}$ and difference operator $Q_{1}$ are defined by:

(6) $$Q_{0}\left(x\right):=\left(\frac{x_{2i-1}+x_{2i}}{2}\right),i=1,2,\cdots ,2^{n-1},$$

(7) $$Q_{1}\left(x\right):=\left(\frac{x_{2i-1}-x_{2i}}{2}\right),i=1,2,\cdots ,2^{n-1},$$

where the operator $Q_{0}$ and $Q_{1}$ are the low and high pass filters of the Harr wavelet [28], respectively. For simplicity, $Q_{0}$ and $Q_{1}$ can be written in matrix form:

(8) $$Q_{j}={\left(\begin{array}{ccccccc} \frac{1}{2} & \frac{{\left(-1\right)}^{j}}{2} & 0 & 0 & \cdots & 0 & 0\\ 0 & 0 & \frac{1}{2} & \frac{{\left(-1\right)}^{j}}{2} & \cdots & 0 & 0\\ 0 & 0 & 0 & 0 & \cdots & \frac{1}{2} & \frac{{\left(-1\right)}^{j}}{2} \end{array}\right)}_{2^{n-1}\times 2^{n}},j=1,2.$$

**Figure 1:**
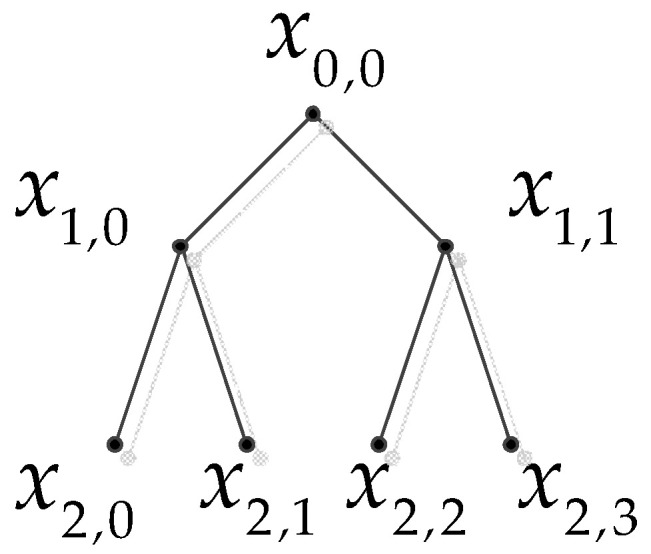
Hierarchical decomposition of x (scale = 3).

(2) Let e be a nonnegative integer and $L_{k}$ equals to 0 or 1, where k=1,2,⋯,n. For a given e, there is a unique vector $\left[L_{1},L_{2},\cdots ,L_{n}\right]$ that fulfills Equation (9). Then, the hierarchical decomposition of a time series can be defined by Equation (10):

(9) $$e=\sum_{k=1}^{n}L_{k}2^{n-k},$$

(10) $$x_{n,e}=Q_{L_{n}}\circ Q_{L_{n-1}}\circ \cdots Q_{L_{1}}\left(x\right),$$

where $x_{n,e}$ denotes the hierarchical component (i.e., the subsequence) of the original time series. To illustrate the decomposition process more clearly, the hierarchical components can be arranged in a tree diagram (see Figure 1). In Figure 1, the original time series is represented as $x_{0,0}$ at the root node. After an average and difference operation, the root node $x_{0,0}$ has a left child node $x_{1,0}$ and a right child node $x_{1,1}$, which correspond to the lower and higher frequency components of $x_{0,0}$, respectively. Analogously, each node $x_{n,e}$ has the left child node $x_{n+1,2e}$ and the right child node $x_{n+1,2e+1}$. In fact, nodes $x_{0,0}$, $x_{1,0}$ and $x_{2,0}$ are equal to the coarse-graining process (which is the multiscale decomposition method used in MSE) at scale 1, 2 and 4, respectively. In other words, the hierarchical decomposition not only preserves the advantages of coarse-graining, but also additionally focuses on the higher frequency components in diverse scales. Hence, it is able to provide more information of the time series than coarse-graining.

### 2.3. Hierarchical Cosine Similarity Entropy

Within the HCSE algorithm, only the hierarchical decomposition is required to proceed prior to entropy estimation. Then, each subsequence at the node is served as an input of CSE to measure its complexity.

## 3. Parameters Selection for HCSE

To compute the HCSE algorithm, parameters such as tolerance $r_{CSE}$, embedding dimension m, data length N and scale factor s must be properly selected. The selection of these parameters is studied in the next subsections using uncorrelated random noise and long-term correlated noise: the White Gaussian noise (WGN) and the 1/f noise. Because time lagτ is analogous to down sampling to some extent, it is typically set as τ=1 for structural preservation [29].

### 3.1. Selection of Tolerance $r_{CSE}$

**Figure 2:**
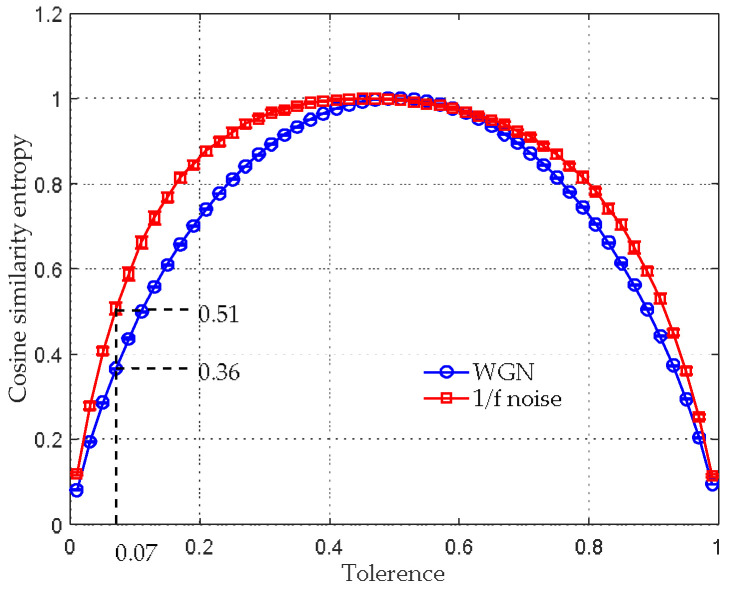
Selection of tolerance for CSE.

Notice that the range of angular distance is from 0 to 1, the boundary values of $r_{CSE}$ should also be 0 to 1. We varied $r_{CSE}$ from 0.01 to 0.99 with a step length of 0.02 to observe how CSE values change against $r_{CSE}$ (see Figure 2). The results in Figure 2 were obtained by 30 independent trials, in which the embedding dimension, time lag and data-length were chosen as the recommended m=2, τ=1 and N=10,000, respectively [20,29]. It can be seen that the mean CSE values of both WGN and 1/f noise are firstly increased with an increase in $r_{CSE}$ from 0.01 to 0.49, and then decrease as the $r_{CSE}$ increases from 0.51 to 0.99. By comparing the mean CSE values of WGN with 1/f noise, it is found that they are more discriminative between $r_{CSE}=0.07\sim 0.21$. Therefore, the tolerance should be selected within the range, in this paper, $r_{CSE}=0.07$ is chosen for subsequent analysis.

### 3.2. Selection of Embedding Dimension m

**Figure 3:**
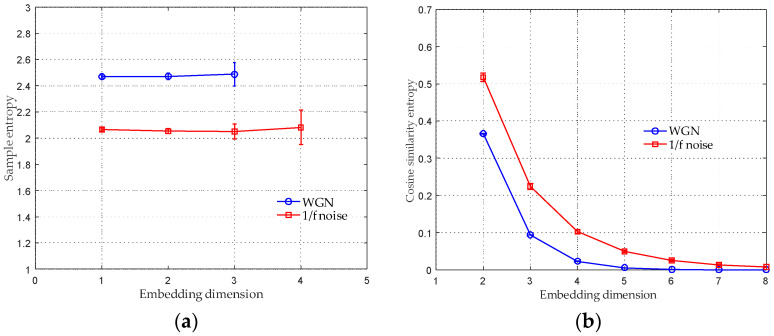
The mean entropy values with their SD error bar over a varying embedding dimension. (**a**) the results of SE; (**b**) the results of CSE.

In this subsection, the relation of CSE values and a varied m is studied by conducting 30 independent trials. $r_{CSE}=0.07$, τ=1 and N=10,000 were selected to calculate the CSE. For comparison, the SE of WGN and 1/f noise were also computed with diverse m. Parameters for computing SE were chosen as m=2, τ=1 and $r_{SE}=0.15\cdot \rho$ [16,17,18,19], where ρ denotes the standard deviation (SD) of the analyzed time series. The average entropy values with their SD error bar over a varying embedding dimension are shown in Figure 3. Figure 3a provides the results of the SE in which the mean SE values remain constant for different m and the SD of SE values increases as the m increases. It can be seen that only a small range of m=[1,2,3] and m=[1,2,3,4] were plotted for the WGN and the 1/f noise, respectively. This is because that the SE algorithm produces undefined entropy beyond the above mentioned range.

In Figure 3b, the mean CSE values of both synthetic signals decrease as m increases. They approach to 0 when m≥6. In addition, the SD of entropies remains constantly small. The results in Figure 3b can be explained by the conclusion in [30], that is, as the embedding dimension m increases, the trajectory of phase-space tends to be more and more deterministic, meaning that lower and lower complexity. The situation m=1 is not given in Figure 3b, this is because that the angular distance is valid for vectors with at least two elements. Therefore, m=2 is the minimum embedding dimension for calculating the CSE. By comparing Figure 3a with Figure 3b, it can be seen that the CSE algorithm can provide more stable entropy estimation in a broader range of m than SE. Since the CSE value approaches to 0 for a large m, a smaller embedding dimension, such as m=2 and m=3, is recommended to compute the algorithm. We selected m=2 for subsequent calculation.

### 3.3. Selection of Data-Length N

**Figure 4:**
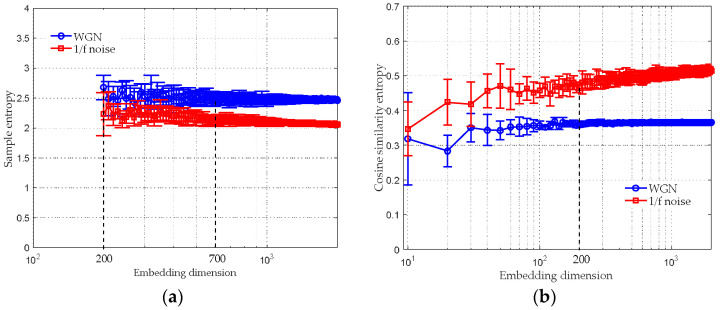
The mean entropy values with their SD error bar over a varying data-length. (**a**) the results of SE; (**b**) the results of CSE.

We examined the relationship between CSE values and data-length in this subsection. For comparison, the SE was also applied to compute the same synthetic signals. Parameters for computing the SE and the CSE were selected as m=2, τ=1, $r_{SE}=0.15\cdot \rho$ and $r_{CSE}=0.07$. The average entropy values with their SD error bar over a varying data-length N are plotted in Figure 4. The data-length N was varied from 10 to 2000 with a step length of 10. In Figure 4a, because of producing undefined entropy, the SE of the WGN and the 1/f noise are invalid when N≤200; when 200≤N≤700, both synthetic signals acquire unstable entropy estimates with a large SD; their entropies become stable when N≥700. The results in Figure 4a correspond well to that in [21], where it is shown that the SE algorithm requires sufficient samples.

In Figure 4b, the CSE algorithm is valid even for 10 samples; when 100≤N≤200, the CSE values of both synthetic signals have a large SD; their entropy estimates become stable when N≥200. Comparing Figure 4a with Figure 4b, it can be found that the WGN and the 1/f noise are more distinguishable by employing the CSE rather than the SE. In contrast to SE, the CSE is more stable in processing short-time series. Regarding the issue of selecting proper data-length N for computing the CSE, it should be chosen according to actual needs as long as it is larger than 200.

### 3.4. Selection of Scale Factor s

It is also necessary to determine the scale factor s appropriately. There is a doubling reduction in the data-length when the scale factor increases by 1. Therefore, the selected s has to ensure that each subsequence has a data-length larger than 200 (as discussed in Section 3.3). Without loss of generality, s=5 was chosen for multiscale analysis in the subsequent study.

## 4. Feature Extraction of Synthetic Signals and Real Ship-Radiated Noise

We applied the proposed HCSE to analyze synthetic signals and real ship-radiated noise, the MSE was also utilized to compute the same signals for comparison. Parameters for computing the HCSE and the MSE were set as m=2, τ=1, $r_{SE}=0.15\cdot \rho$, s=5 and $r_{CSE}=0.07$. Considering that the hierarchical decomposition demands a data-length of $N=2^{n}$, the data-length N was selected as 8192.

### 4.1. HCSE Analysis for Synthetic Signals

It is necessary to firstly apply the proposed HCSE method to analyze signals with known characteristics and complexity levels. In this subsection, the uncorrelated WGN and the long-term correlated 1/f noise were analyzed. The results are obtained from 30 independent realizations.

**Figure 5:**
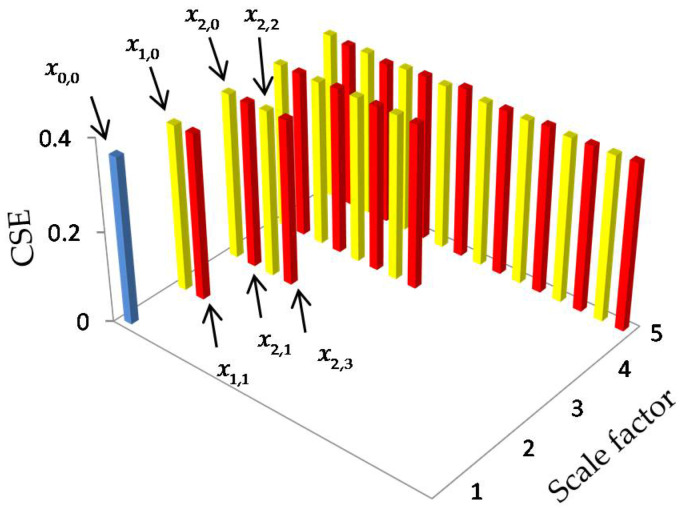
HCSE analysis of the WGN. (the yellow bar and the red bar, respectively, represent the lower and the higher frequency components, as is the same for the subsequent results).

Figure 5 offers the mean HCSE values of WGN. It is shown that the mean HCSE value of every node is approximately equal to 0.365, implying that each subsequence is as complex as the original WGN.

**Figure 6:**
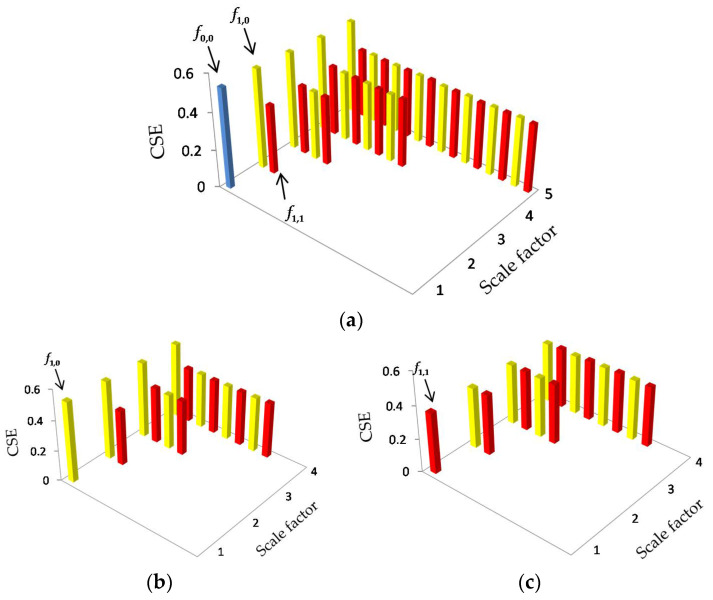
HCSE analysis of the 1/f noise. (**a**) The results of original 1/f noise; (**b**) the results of $f_{1,0}$; (**c**) the results of $f_{1,1}$.

Figure 6 depicts the HCSE analysis results of 1/f noise. In Figure 6, the 1/f noise is denoted by $f_{0,0}$, while its lower frequency components and higher frequency components at scale 2 are represented by $f_{1,0}$ and $f_{1,1}$, respectively. In Figure 6a, as the scale factor s increases, the mean HCSE values of node $f_{s-1,0}$ remain constant at 0.517, while the other hierarchical components have an equal HCSE value of 0.365, which is equal to the WGN. The subtrees with root node $f_{1,0}$ and $f_{1,1}$ are also plotted in Figure 6b,c, respectively. Comparing Figure 5 with Figure 6c, it can be observed that the subtree of $f_{1,1}$ looks pretty much like that of the WGN. Similar results can also be found by comparing Figure 6a,b. Hence, Figure 5 and Figure 6 verifies the assumption in [28] that $f_{1,0}$ is still 1/f noise, while $f_{1,1}$ is approximately equal to the WGN.

**Figure 7:**
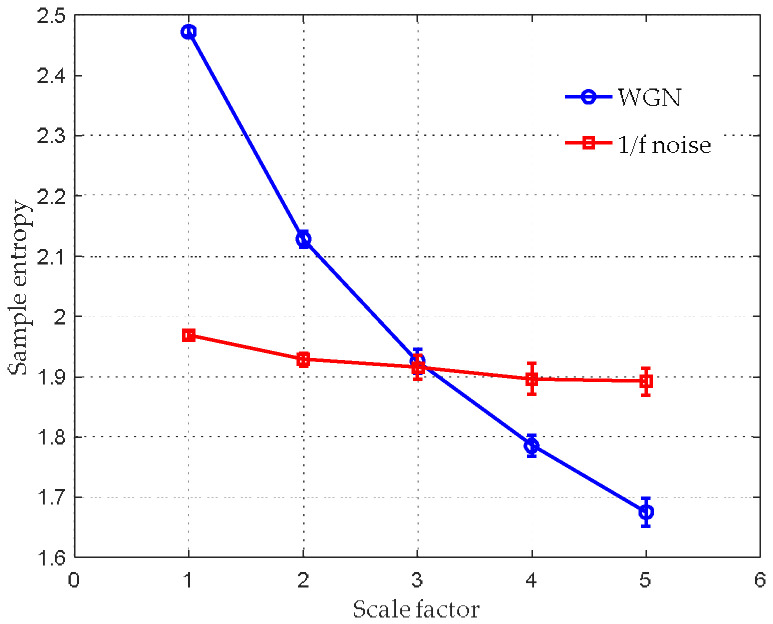
MSE analysis for synthetic signals.

The average SE values with their SD error bar over a varying scale factor are shown in Figure 7. The WGN achieves the highest mean entropy value at scale 1. The entropy curve decreases as the scale factor increases. Its SE value finally falls to 1.68 when s=5. With respect to the 1/f noise, the mean entropies remain constant between 1.89 and 1.98 for the whole range of scale factors.

Comparing the MSE analysis result with that of the HCSE, it is shown that HCSE can provide information of the higher frequency components of a signal, which is not provided by MSE. Hence, HCSE is capable of extracting features of a signal more comprehensively and precisely. Furthermore, the HCSE analysis result of the WGN corresponds well with the fact that the WGN is not structurally complex and it also agree well with previous claim in [28] that different hierarchical components of WGN are still WGN.

### 4.2. Feature Extraction of Real Ship-Radiated Noise

**Figure 8:**
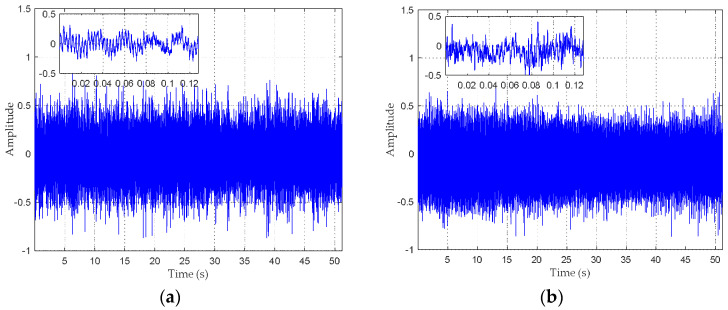

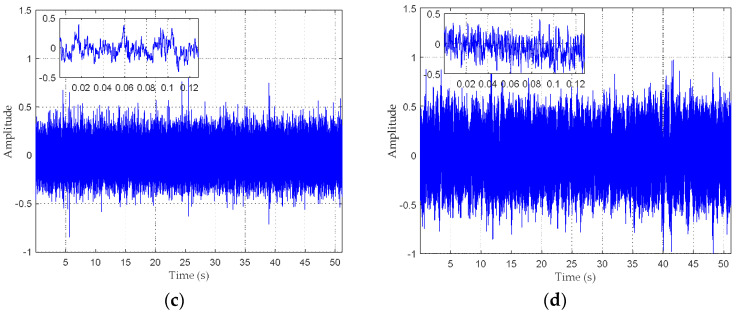
Recordings of four types of ship-radiated noise. (**a**) type A; (**b**) type B; (**c**) type C; (**d**) type D.

**Figure 9:**
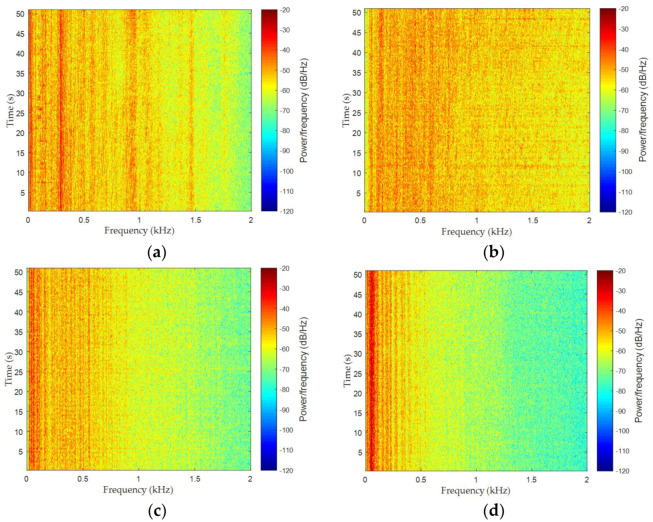
Spectrograms of four types of ship-radiated noise. (**a**) type A; (**b**) type B; (**c**) type C; (**d**) type D.

We utilized the proposed HCSE to extract features of four types of ship radiated noise, which were recorded in the South China Sea. The depth of the experimental area is about 4000 m, and the seabed is approximately flat. The data acquisition was carried out under the level 1 sea state to avoid serious influence of ocean ambient noise. The sensitivity and frequency response of the omnidirectional hydrophone are 170 dB re 1 v/μpa and 0.1 Hz–80 kHz, respectively. The hydrophone, which was carried by a research ship, was deployed at a depth of 30 m. In order to eliminate the self noise of the research ship, its engines were shut down and its speed reduced to approximately zero. Then, four different target ships, which were 2.5 km away from the research ship, moved towards the hydrophone at an average speed of 10 knots. When one target ship was moving, the other ships were rested. When its distance to the hydrophone is less than 1 km, the target ship would slow down and stop. The data was recorded at a sampling rate of 16 kHz. It should be pointed out that four target ships have different size, tonnage and propulsion equipment, so that they can be classified into four categories. In the subsequent study, the radiated noise of four different target ships are represented as type A, B, C and D, respectively. Each type contains 819,200 sample points, which are cut equally into 100 pieces for analysis. Figure 8 depicts the normalized waveforms of four types of ship-radiated noise. In order to show more details, detail view is also provided in each picture. Spectrograms of four types of ship-radiated noise are given in Figure 9, which represents the energy distribution against time and over frequencies, the amount of acoustic power is represented as the intensity at each time frequency point. The spectrogram is a method to recognize diverse vessels, because different types of ships may have different acoustic energy distribution against frequencies. It is found that type A and B can be distinguished well, since type B have obvious higher energy in high frequency area (1.5–2 kHz). However, the acoustic energy signatures of type C and D are too similar to discriminate them. Hence, it is necessary to extract other features of the ship, such as entropy.

**Figure 10:**
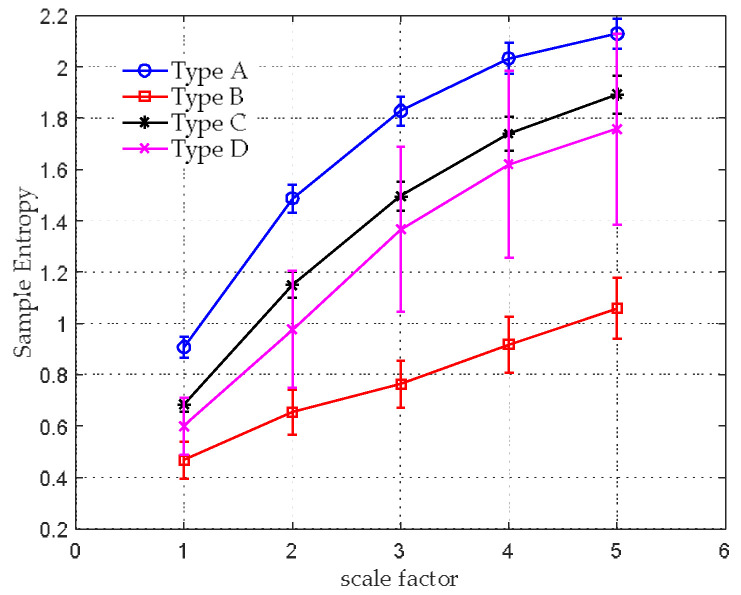
Feature extraction results of the MSE.

The feature extraction results of MSE are shown in Figure 10 and Table 1. The average SE values with their SD error bar over a varying scale factor are plotted in Figure 10. It can be seen that, for all four types, there is an increasing mean SE values as the scale factor increases. The MSE features seem to be effective for classifying type A, B and C, because their entropies over diverse scales are visually and statistically discernable. However, type C and D have a similar entropy distribution over different scales, and the SE estimation for type D is unstable, they may not be distinguished well by using MSE.

**Figure 11:**
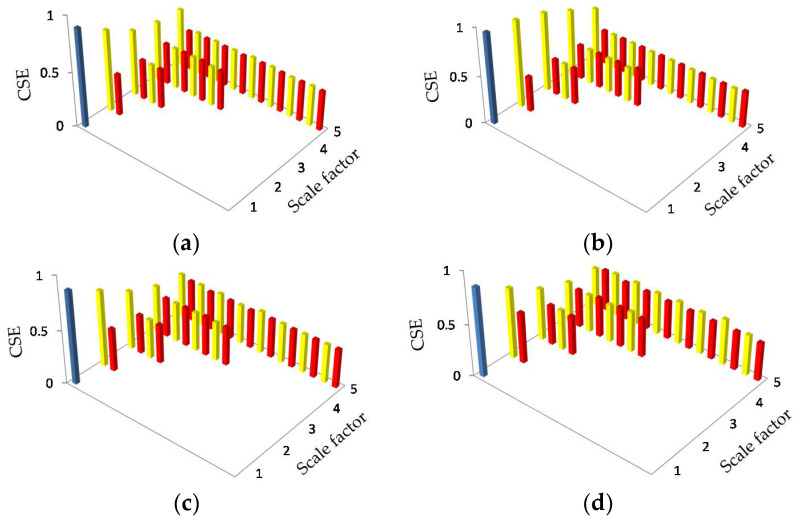
HCSE analysis for real ship-radiated noise.

Figure 11, Table 2 and Table 3 provide the HCSE feature extraction results. Unlike MSE, the HCSE results in decreasing entropies at nodes $x_{s-1,0}$ with an increasing scale factor. As mentioned before, the hierarchical components at nodes $x_{s-1,0}$ are equal to the coarse-grained subsequences at scale $2^{s-1}$. Thus, similar with the MSE analysis results, type C and D can not be recognized well by only observing the CSE values at nodes $x_{s-1,0}$. Fortunately, except for the lower frequency components, the HCSE is able to provide information of the higher frequency components of a signal. Comparing Figure 11 with Figure 5, it is seen that the subtrees with root node $x_{1,1}$ of type A, B, and C (i.e., the bold represented parts in Table 2 and Table 3) look pretty much like that of the WGN, where the CSE values of every node is approximately equal to 0.365. In the corresponding area, type D achieves obvious higher entropies at nodes $x_{1,1}$, $x_{3,6}$, $x_{4,8}$, $x_{4,10}$, $x_{4,12}$ and $x_{4,14}$, where the CSE values are larger than 0.39. The result means that type D has a more complex structure than the other 3 types. Type C and D are distinguishable by comparing entropies in that area.

### 4.3. Feature Classification

To test the validity of the proposed algorithm, the extracted features were input into the widely used probability neural network (PNN) [31] for training and testing. 20 pieces of ship-radiated noise were set as training samples and the other 80 pieces were used for testing. As shown in Table 4 and Table 5, the classification results agree well with the feature extraction results in Section 4.2. For example, the MSE can perfectly classify type A, B and C, but no type D is correctly recognized, and the classification accuracy turns out to be 75%. Compared with MSE, HCSE achieves a little lower accuracy in classifying type A, but the classification performance of type D is remarkably improved. The classification accuracy finally reaches 95.63%, which is 20.63% higher than MSE.

## 5. Conclusions

The classification performance of passive sonar can be improved by extracting the features of ship-radiated noise. Traditional feature extraction methods neglected the nonlinear features in ship-radiated noise, such as entropy. For the purpose of extracting useful nonlinear features of ship radiated noise, the HCSE method is proposed in this paper. The presented algorithm takes strength of both hierarchical decomposition and CSE. The advantages of the proposed method are as follows from the simulation and experimental results:

(1) The undefined entropy is unlikely to occur in HCSE by utilizing Shannon entropy rather than conditional entropy and employing angular distance instead of Chebyshev distance. As a consequence, the HCSE method is valid when data-length N=10, while the MSE method is invalid when N≤200.
(2) The HCSE is suitable for short time series. It can provide stable entropy estimation when N≥200, while the MSE demands N≥700.
(3) The HCSE analysis result of the WGN is in consistent with the fact that WGN is not structurally complex, and it also agrees well with claim that hierarchical components of WGN are still WGN.
(4) The HCSE method can extract the features of a signal more comprehensively and precisely, because it takes both lower and higher frequency components into consideration. Compared with MSE, the classification accuracy of real ship-radiated noise is significantly improved from 75% to 95.63% by using HCSE.

## Figures and Tables

**Table 1:** Feature extraction results of the MSE.

Type	Mean SE Values				
	Scale 1	Scale 2	Scale 3	Scale 4	Scale 5
A	0.907	1.486	1.827	2.03	2.130
B	0.467	0.654	0.764	0.917	1.058
C	0.683	1.150	1.496	1.738	1.893
D	0.599	0.977	1.366	1.620	1.757

**Table 2:** Mean HCSE values for type A and B.

Type	Scales					Type	Scales				
	1	2	3	4	5		1	2	3	4	5
A	0.896	0.743	0.604	0.548	0.534	B	0.956	0.920	0.836	0.702	0.566
		**0.380**	0.366	0.380	0.364			**0.372**	0.383	0.370	0.363
			**0.365**	0.376	0.386				**0.385**	0.366	0.365
			**0.366**	0.376	0.370				**0.386**	0.371	0.364
				**0.378**	0.381					**0.370**	0.365
				**0.379**	0.370					**0.376**	0.364
				**0.366**	0.376					**0.370**	0.364
				**0.366**	0.365					**0.402**	0.366
					**0.387**						**0.367**
					**0.372**						**0.365**
					**0.380**						**0.364**
					**0.370**						**0.367**
					**0.364**						**0.363**
					**0.364**						**0.363**
					**0.370**						**0.367**
					**0.364**						**0.386**

**Table 3:** Mean HCSE values for type C and D.

Type	Scales					Type	Scales				
	1	2	3	4	5		1	2	3	4	5
C	0.870	0.714	0.553	0.447	0.411	D	0.856	0.680	0.504	0.401	0.378
		**0.402**	0.370	0.371	0.384			**0.489**	0.385	0.368	0.400
			**0.370**	0.370	0.391				**0.389**	0.367	0.411
			**0.366**	0.370	0.365				**0.377**	0.385	0.371
				**0.370**	0.392					**0.367**	0.417
				**0.374**	0.367					**0.387**	0.373
				**0.365**	0.368					**0.394**	0.407
				**0.365**	0.362					**0.379**	0.370
					**0.398**						**0.417**
					**0.368**						**0.374**
					**0.370**						**0.410**
					**0.362**						**0.371**
					**0.363**						**0.442**
					**0.363**						**0.373**
					**0.363**						**0.392**
					**0.365**						**0.367**

**Table 4:** PNN classfication results of MSE.

Type	Recognized as				Accuracy
	A	B	C	D	
A	80	0	0	0	100%
B	0	80	0	0	100%
C	0	0	80	0	100%
D	0	15	65	0	0%
In total	80	95	145	0	75%

**Table 5:** PNN classfication results of HCSE.

Type	Recognized as				Accuracy
	A	B	C	D	
A	69	0	11	0	86.25%
B	0	80	0	0	100%
C	0	0	80	0	100%
D	1	0	2	77	96.25%
In total	70	80	93	77	95.63%
